# Steering Demands Diminish the Early-P3, Late-P3 and RON Components of the Event-Related Potential of Task-Irrelevant Environmental Sounds

**DOI:** 10.3389/fnhum.2016.00073

**Published:** 2016-03-01

**Authors:** Menja Scheer, Heinrich H. Bülthoff, Lewis L. Chuang

**Affiliations:** ^1^Department of Perception, Cognition and Action, Max Planck Institute for Biological CyberneticsTübingen, Germany; ^2^Department of Cognitive and Brain Engineering, Korea UniversitySeoul, South Korea

**Keywords:** steering, mental workload, distraction, MMN, early P3, late P3, RON

## Abstract

The current study investigates the demands that steering places on mental resources. Instead of a conventional dual-task paradigm, participants of this study were only required to perform a steering task while task-irrelevant auditory distractor probes (environmental sounds and beep tones) were intermittently presented. The event-related potentials (ERPs), which were generated by these probes, were analyzed for their sensitivity to the steering task’s demands. The steering task required participants to counteract unpredictable roll disturbances and difficulty was manipulated either by adjusting the bandwidth of the roll disturbance or by varying the complexity of the control dynamics. A mass univariate analysis revealed that steering selectively diminishes the amplitudes of early P3, late P3, and the re-orientation negativity (RON) to task-irrelevant environmental sounds but not to beep tones. Our findings are in line with a three-stage distraction model, which interprets these ERPs to reflect the post-sensory detection of the task-irrelevant stimulus, engagement, and re-orientation back to the steering task. This interpretation is consistent with our manipulations for steering difficulty. More participants showed diminished amplitudes for these ERPs in the “hard” steering condition relative to the “easy” condition. To sum up, the current work identifies the spatiotemporal ERP components of task-irrelevant auditory probes that are sensitive to steering demands on mental resources. This provides a non-intrusive method for evaluating mental workload in novel steering environments.

## Introduction

Safety concerns have strongly motivated research in determining the demands, or workload, that users experience while performing closed-loop steering tasks, particular in the context of driving a car or piloting an aircraft (for a general review about workload, see Kramer, 1991; Wickens, 2008; Young et al., 2015). Even if competence can be maintained in spite of high mental workload, such scenarios leave little spare capacity for handling unexpected occurrences. There is no doubt that steering places high requirements on visual and motoric resources (Land and Lee, 1994; Salvucci and Gray, 2004). Besides this, some aspects of steering have also been shown to require mental resources (Wickens et al., 1983, 1984). This has been typically demonstrated with the use of dual-task paradigms that induce a competition for mental resources between the primary steering task and an appropriately chosen secondary task (McLeod, 1977; Wickens and Gopher, 1977). The purpose of this article is to evaluate the demands that steering places on mental resources without requiring the user to perform a secondary task. To do so, we investigate how steering demands modify the event-related potentials (ERPs) to task-irrelevant auditory probes. The steering task is further manipulated for two aspects of steering that are known to influence handling difficulty, namely the bandwidth of disturbance and the complexity of (vehicle) control dynamics.

Workload can be defined as the ratio between the demands of a task and the resources of the human operator. Its concept originates from the idea that human operators possess, at any given time, a limited reserve of mental resources (Kramer, 1991; Wickens, 2008). By introducing a competition for this limited reserve, for example by requiring participants to perform two tasks simultaneously, researchers are able to investigate how difficulty manipulations in a primary task can create a demand for resources that are drawn away from an accompanying secondary task. Changes in resource demands are indexed by secondary task performance. A comparison of performance measures on competing tasks typically demonstrate that participants are capable of varying the relative prioritization of competing tasks (Wickens and Gopher, 1977), but only when the tasks overlap in their resource requirements (McLeod, 1977). The “Multiple Resource Theory” provides a framework that allows researchers and practitioners to define the resource requirements of different tasks and in doing so, predict possible conflicts (Wickens and Yeh, 1983; Wickens, 2002, 2008). Within this framework, a steering task places obvious demands on visual perception and motoric responses. By using electroencephalography (EEG) to measure the ERP to secondary task stimuli, Wickens and colleagues were able to demonstrate the demands of various aspects of steering on mental resources as well.

To date, ERP studies have broadly demonstrated that steering demands tend to reduce the amplitude of the P300, an ERP component that is generated by the target stimuli of a secondary task (e.g., Wickens et al., 1977; Isreal et al., 1980; Wickens and Yeh, 1983). Dual-task studies that investigate steering demands typically require participants to detect and explicitly respond to infrequently presented “oddball” targets as a secondary task. “Oddballs” elicit a prominent P300 component in the EEG signal. The P300 is a positive deflection between 250–400 ms and its amplitude has been used to index the level of experienced workload (Kok, 1997). The finding that steering demands diminish P300 amplitudes in an accompanying “oddball” detection task is commonly interpreted as follows. The primary steering task places prioritized demands on mental resources, resulting in the reduced availability of mental resources that would otherwise be recruited for the detection of secondary “oddball” targets (Wickens et al., 1977; Isreal et al., 1980; Wickens and Yeh, 1983). Hence, the reduced availability of mental resources is reflected in the reduced amplitudes of P300 that are elicited by the detected “oddballs”. This serves as a proxy for evaluating the demands for mental resources, given different manipulations of steering difficulty. Some steering parameters exert a uniform cost on P300 amplitudes regardless of their manipulated difficulty levels, while increasing the difficulty levels of other parameters can induce decreased P300s to secondary “oddball” targets. For example, increasing the number of simultaneously tracked dimensions (Wickens et al., 1977; Kramer et al., 1983; Sirevaag et al., 1989), tracking speed (Kida et al., 2004), and the frequency bandwidth of the tracked target (Isreal et al., 1980) do not result in a decrease of P300 amplitudes. In contrast, increasing the complexity of control dynamics (e.g., from a first-order to a second-order integrator; Wickens et al., 1983, 1984; Sirevaag et al., 1989) or the unpredictability of the tracked target (Kida et al., 2004) result in corresponding decreases in P300 amplitudes. Other ERP components have also been analyzed for their sensitivity to changes in steering demands, albeit with mixed results. Kida et al. (2004) reported a decrease in the amplitude of the N140 component to the somatosensory targets of a secondary oddball task, which did not vary with the predictability of the steering task.

Until now, ERP studies of steering demands have mainly been performed in the presence of a secondary task that contains the stimuli for eliciting the ERP. It is generally believed that ERP probes are only effective for evaluating the resource demands of tasks that they are in explicit conflict with. Indeed, Wickens et al. (1983) have shown that the influence of steering demands on P300 amplitudes is removed when the ERP probes were task-irrelevant. Unfortunately, dual-task paradigms present several limitations in understanding steering demands. First, requiring an overt response to a secondary task interferes with the performance of the primary steering task (Wickens et al., 1983). In this regard, the secondary task is not a passive consumer of residual mental resources but is, rather, in direct competition with the primary task for shared resources. Second, the researcher has little control over how participants might choose to divide their resources between primary and secondary task, regardless of explicit instructions. Finally, estimated workload from ERP measurements could be due to the interaction of the primary and the secondary task demands, instead of the primary task alone. These reasons, amongst others, have motivated the development of non-intrusive methods for estimating primary task demands that do not necessitate a secondary task.

In contrast to Wickens et al.’s (1983) findings, ERPs to task-irrelevant stimuli can sometimes be demonstrated to vary with the demands of a task that is performed in isolation. This has been shown with the use of ERP probe stimuli that are more likely to recruit larger momentary shifts of resources than simple beep tones, such as complex environmental sounds (Courchesne et al., 1975; Ullsperger et al., 2001; Polich, 2003). Such stimuli are task-irrelevant and reliably elicit a positive ERP component termed the novelty-P3 (P3a)—that has a similar time-course to the P300 but with a frontal instead of a parietal distribution (Polich, 2007). Given their task-irrelevant nature, it is more reasonable to assume that their elicited ERP components reflect residual resources that are not consumed by the demands of the investigated task. Task-irrelevant probes have been used to estimate the demands of a variety of tasks including arithmetic and visual monitoring (Ullsperger et al., 2001), working memory task (i.e., *n*-back task; SanMiguel et al., 2008), Tetris^®^ (Miller et al., 2011; Dyke et al., 2015), first-person-shooter (Allison and Polich, 2008) and car racing games (Burns and Fairclough, 2015). It has not always been necessary to employ novel environmental sounds in order to generate ERPs for the evaluation of task demands—simple beep tones have proven to be sufficient in some instances (Burns and Fairclough, 2015). Nonetheless, there are also other examples whereby simple beep tones do not generate ERPs (i.e., P3a) that are sensitive to task demands (e.g., Ullsperger et al., 2001; Muller-Gass et al., 2007). Environmental sounds have the added value of generating larger novelty-P3s that are further separable for an early and late P3 component, which are claimed to be functionally distinct (Alho et al., 1998; Yago et al., 2003; McDonald et al., 2010). Early P3 is claimed to reflect post-sensory detection of unexpected events that contradict the observer’s representation of the external world, while late P3 is claimed to reflect attentional processing of the unexpected event. Besides novelty-P3, other ERP components of task-irrelevant probes (i.e., N1/MMN; Ullsperger et al., 2001; Dyke et al., 2015; P2 and N2; Allison and Polich, 2008; late positive potential or LPP; Miller et al., 2011) have also been claimed to be diminished by increased task demands, albeit less consistently.

Taken together, ERP probes can be regarded as distractors that demand resources either through explicit competition with the primary task (Isreal et al., 1980; Wickens et al., 1983, 1984) or by implicitly drawing upon residual resources that are unconsumed by the primary task (SanMiguel et al., 2008; Miller et al., 2011; Burns and Fairclough, 2015; Dyke et al., 2015). Previous work that assessed steering demands might have required ERP probes to be task-relevant because the employed probes (i.e., beep tones) did not recruit sufficient resources to indicate the influence of steering demands.

ERP components that are elicited by distracting stimuli have been suggested to reflect three stages of distraction (Schröger and Wolff, 1998; Escera and Corral, 2007; Wetzel and Schröger, 2014). Based on the specific ERP components that are decreased with an increase of the task demands, inferences about the stages of distraction that are influenced can be drawn. The first stage of distraction is the detection that the model of the environment was violated. When engaged in a task, participants can be expected to be primarily focused on this task. At the same time, the regularities of the acoustic environment are encoded and used to form a predictive model of the surroundings. Whenever a current event violates this predictive model, the distraction process is initiated. This first stage of distraction is reflected in the elicited ERP by the mismatch negativity (MMN). The MMN is an early, negative ERP component that is apparent in the difference wave between the distractor- and the standard stimuli, for example in an oddball paradigm. Thus, the presence of a MMN indicates early sensory detection of an unexpected change in the environment. The second stage is the, voluntary or involuntary, orientation of attention towards the distracting event. Depending on the level of readily available resources and the eliciting event, resources might be directed towards the distracting event in order to process it. This stage is reflected by the occurrence of the novelty-P3 component. The third stage describes a disengagement of resources from the distracting event and a re-orientation back to the task at hand. Disengagement from the distractor stimuli is reflected by the re-orientation negativity (RON), a late negative component.

The current study investigates the influence of steering demands on ERP components that are generated by task-irrelevant auditory distractor stimuli. In the viewing baseline condition, we expect distractor stimuli to elicit ERP components that correspond to the three-stage distraction model, regardless of whether they are infrequently presented beep tones or infrequently presented environment sounds. However, we expect these ERP components to be larger when generated by environment sounds. Furthermore, we expect these ERP components to decrease when participants are required to perform a steering task, but only when they are generated by environmental distractors. We employ a data-driven approach (i.e., mass univariate analyses; Groppe et al., 2011) to ensure the validity of any correspondence between distractor ERP components and steering demands. This approach allows us to define each affected component in terms of its spatial and temporal characteristics, as opposed to restricting our analyses to an *a priori* selection of components (cf., Miller et al., 2011; Dyke et al., 2015). ERP components that are found to be sensitive to steering demands are subsequently submitted for permutation tests to evaluate their suitability for discriminating between manipulated levels of steering difficulty. We manipulate steering difficulty by either increasing the frequency bandwidth of the disturbance that is experienced during steering (cf., Isreal et al., 1980), or by varying the complexity of the control dynamics (cf., Wickens et al., 1983). We expect more participants to demonstrate a significant reduction in these targeted ERP components in the “hard” condition compared to the “easy” condition.

## Materials and Methods

### Participants

We tested 24 right-handed volunteers (seven women, mean age = 27.9 years, *SD* = 5.2). All participants reported normal or corrected-to-normal vision, no hearing impairment and no history of neurological diseases. The experimental procedure was approved by the MPG Ethics Council and all participants gave written informed consent.

### Stimuli and Apparatus

The experiment was set up in a dimly-lit, low noise environment. It consisted of a primary steering task and the presentation of task-irrelevant, auditory stimuli. The steering task was presented via a central display (1027 × 581 mm, resolution 1920 × 1080 px), approximately 180 cm away from the seated participants. Auditory stimuli were presented to both ears via headphones (MDR-CD380, Sony), that where driven by a soundcard (sampling frequency: 96 kHz; DELTA1010LT, M-Audio). A secondary heads-down display informed the participants of their most recent steering performance and the current experimental status. Data collection was performed, using customized software, written in Matlab Simulink. The software version of the NASA-TLX questionnaire (Hart and Staveland, 1988) was presented on a separate notebook.

Two lines (length: 16° visual angle, thickness: 2 px) were presented on a blue background. These lines were a white horizontal non-moving reference line and a second black line that rotated around the joint center of both lines. A right-handed sidestick (Extreme 3D Pro, Logitech) with a spring constant of 0.6 N/degree was used as input device.

During the entire experiment, participants were probed with task-irrelevant stimuli with a random inter-stimulus interval (mean = 1.20 s, *SD* = 62 ms). Infrequently presented environmental sound distractors (prob. of presentation: *p* = 0.1) were intermixed with frequent, standard (*p* = 0.8) and infrequent distractor (*p* = 0.1) beep-tones. Two easily discriminable beep-tones were used (i.e., 300 and 700 Hz) and their probability (*p* = 0.1 and *p* = 0.8) was counter-balanced across participants. The environmental sounds consisted of a set of 30 recognizable complex sounds (e.g., human laughter) that were selected from a database obtained from the New York State Psychiatric Institute (Fabiani et al., 1996). The environmental sounds were presented in quasi-random order without replacement. Environmental sounds, as well as standard and distractor beep-tones, had a mean duration of 336 ms (*SD* = 62.5 ms) and a mean intensity of 60 dB SPL (*SD* =0.31 dB). Both, environmental and beep sounds were always preceded by at least one standard beep.

### Task

**Figure 1:**
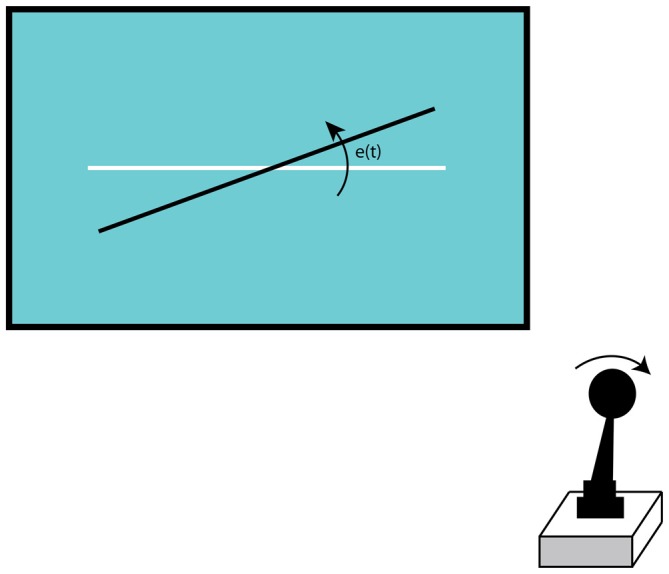
**The steering task required the participants to counteract the quasi-random displacement *e(t)* of the rotating line (black) to the non-moving reference line (white), with lateral sidestick deflections**.

Participants performed a steering task in which they were required to continuously counteract a quasi-random roll motion of a rotating line. This unpredictable roll motion was defined by the forcing function *f_t_(t)* (see Equation (1) and Table 1). Participants were instructed to minimize the displacement *e(t)* of the rotating line (black in Figure 1) relative to the reference line (white in Figure 1), with lateral deflections of the sidestick.

**Table 1:** **Amplitude *A*(*j*), frequency *ω*(*j*) and phase *φ*(*j*) of the ten sine waves, contained in the forcing function, for the “standard”, “easy” and “hard” condition**.

	Standard			Easy			Hard		
j	*A*(*j*) in degree	*ω*(*j*) in rad/s	*φ*(*j*) in rad	*A*(*j*) in degree	*ω* (*j*) in rad/s	*φ*(*j*) in rad	*A*(*j*) in degree	*ω*(*j*) in rad/s	*φ*(*j*) in rad
1	1.34	0.39	2.69	1.36	0.39	3.27	1.33	0.39	2.42
2	1.03	0.83	5.74	0.93	0.83	5.95	1.10	0.83	2.20
3	0.51	1.76	5.72	0.40	1.76	3.95	0.63	1.76	2.35
4	0.26	2.85	5.92	0.19	2.85	3.93	0.34	2.85	4.59
5	0.16	3.90	1.66	0.12	3.90	2.26	0.21	3.90	4.57
6	0.09	5.45	1.53	0.07	5.45	0.59	0.13	5.45	5.67
7	0.06	7.76	1.90	0.05	7.76	1.65	0.08	7.76	0.74
8	0.04	10.50	4.74	0.04	10.50	3.80	0.05	10.50	0.71
9	0.04	13.11	4.06	0.03	13.11	0.15	0.04	13.11	0.21
10	0.03	17.33	4.53	0.03	17.33	4.83	0.03	17.33	3.39

Task-irrelevant sounds were presented that our participants were instructed to disregard. The experiment consisted of steering as well as of viewing trials. The viewing trials presented the same visual feedback in all sessions and served as a baseline. In this condition, participants viewed the steering task that was prerecorded. By comparing the steering trials against these viewing trials that both presented the same visualization, we could determine how the demands of the steering task influenced the measured ERPs, independent of the visualization.

Two aspects of the steering task were used to influence the level of workload in the task: (1) the frequency bandwidth of the roll disturbance and (2) the complexity of the internal control dynamics. In every steering trial, one of these aspects was manipulated, leading to two levels of steering task difficulty, namely “easy” and “hard” for each of the two manipulations. The second aspect was kept constant and will be referred to as “standard”, in the following. The objective was to create two levels of workload for independent manipulations (cf. Isreal et al., 1980; Wickens et al., 1984). Details of these manipulations of engagement are given in the following.

#### Manipulation of the Bandwidth of Roll Disturbance

The roll disturbance was designed as a sum of ten sine waves that could be manipulated for the number and intensity of roll reversals by adjusting the frequency bandwidth, such that the “easy” condition presented less power in the higher frequencies, compared to the “hard” condition. The “standard” condition was designed to be an intermediate of these two conditions.

In all conditions, the forcing function was formalized as the sum of ten sine waves that were non-harmonically related, as described in (1):

(1) $$f_{t}(t)=\sum_{j=1}^{10}A(j)\cdot \sin(\omega (j)\cdot t+\phi (j))$$

The amplitude *A(j)*, frequency *ω(j)* and phase *φ(j)* of these 10 sine waves, for the “standard”, the “easy” and the “hard” condition, are given in the Table 1.

The forcing function in the “standard” condition had a variance of 1.61 degree^2^, adapted from Nieuwenhuizen et al. (2013). In the “easy” condition a variance of 1.47 degree^2^ and in the “hard” condition a variance of 1.78 degree^2^ was applied.

To sum up, the “hard” condition presented larger amplitudes in the higher frequencies that resulted in more instances of roll-reversals than the “standard” and “easy” condition.

#### Manipulation of the Control Dynamics

By manipulating the control dynamics, the motion of the rotating line, relative to the sidestick input of the participants, was manipulated. The control dynamics can be formally described as the transfer function *H(s)*.

In the “standard” condition the transfer function had the form of:

(2) $$H_{\text{standard}}(s)=\frac{2.75}{s\;(s+\omega_{b})}$$

This represents a hybrid controller that reacts to the sidestick input with a weighted mixture of velocity and acceleration control. In other words, depending on the frequency of the sidestick input of the participant, either the velocity or the acceleration of the rotating line was influenced. To manipulate the internal control dynamics for difficulty levels, we removed either the velocity or the acceleration component, resulting in either a pure velocity controller with the following form for the “easy” condition:

(3) $$H_{\text{easy}}(s)=\frac{1.5}{s}$$

or a pure acceleration controller with the following form for the “hard” condition:

(4) $$H_{\text{hard}}(s)=\frac{5}{s^{2}}$$

These transfer functions were adopted from Zollner et al. (2010).

Controlling the acceleration has been shown to be more demanding than controlling the velocity (e.g., Wickens et al., 1984; Sirevaag et al., 1989). When the velocity is controlled, the angle of the sidestick translates to the velocity of the controlled line. In this case, keeping the sidestick in the center results in no motion of the controlled line. When the acceleration is controlled instead, keeping the sidestick in the center results in no further acceleration, but the controlled line will maintain its current velocity. Thus, participants have to anticipate the future consequence of their input commands when using a pure acceleration controller.

### Design and Procedure

The experiment consisted of two sessions on 2 separate days, one that contained the manipulation of the bandwidth of the roll disturbance and one that contained the manipulation of the complexity of the control dynamics. Session order was counterbalanced across participants. Each of the two sessions consisted of four blocks that contained three trials each. The four blocks differed in terms of the implemented difficulty (“easy” or “hard”). Each block contained two steering and one viewing trial, where the order of the trials was randomized for every participant. Each of the trials lasted 4 min 26 s and trials were separated by 20 s of rest. During EEG preparation, participants were trained on every difficulty level and for each manipulation for at least one trial. Over the whole course of the experiment, after each trial, participants were presented with their performance (normalized root-mean-square error, nRMSerror) to keep them motivated. At the end of each block, participants were asked to rate their perceived workload in the NASA-TLX questionnaire for each level of difficulty, separately.

### EEG Signal Processing

The EEG was recorded with 26 active g.tec Ag/AgCl electrodes (g.LADYbird, g.tec), mounted in an elastic cap (g.GAMMAcap, g.tec). The electrooculogram (EOG) was recorded from four additional electrodes: at the outer canthi of the left and right eye, and above and below the left eye. All recorded signals were re-referenced off-line to the linked mastoids. The ground electrode was placed at FPz. The signals were amplified in the range between 0 and 2.4 kHz and digitized with a sampling rate of 256 Hz (g.USBamp, g.tec).

Further processing and analysis of the ERP signal was performed with Matlab and the open source Matlab toolboxes EEGLAB (Delorme and Makeig, 2004) and ERPLAB (Lopez-Calderon and Luck, 2014). In the off-line preprocessing, the data was high pass filtered at 1 Hz and low pass filtered at 15 Hz. Second-order Butterworth filters were used for both filters. From the filtered data, epochs from −200 to 1000 ms, relative to the onset of the presented sounds, were extracted. Epochs that showed blink or eye movement characteristics, in any of the electrodes, were rejected. The remaining epochs were averaged for each auditory stimulus type (environmental distractor, beep distractor, standard beep tone) and baseline corrected with reference to the pre-stimulus interval. The statistical analysis of the ERPs was based on the difference wave between ERPs that were elicited by distractors (the beep and environmental distractors, separately) and standards. This difference wave has been also referred to as distraction potential (DP; Escera et al., 2003).

### Statistical Analysis of the ERPs

We adopted a 2-stage approach for analyzing the ERPs elicited by the environmental and beep distractors. First, we employed mass univariate analyses to: (i) determine the ERP components that were elicited by the distractors; (ii) determine the ERP components that differed between the environmental and beep distractors; and (iii) identify and define the spatiotemporal characteristics of ERP components that were significantly reduced during steering, relative to the viewing baseline condition. To perform the mass univariate analyses, measured brain potentials were compared between the relevant conditions at all time points (between 100–900 ms after the presentation of the auditory stimuli) and all measured electrodes (26 electrodes distributed over the scalp). Two-tailed *t*-tests were performed between the compared conditions to yield *t*-values for every time-point of each electrode. The false discovery rate (FDR) was controlled using the Benjamini and Yekutieli (2001) procedure with a FDR level of 5%. This particular FDR procedure guarantees that the true FDR will approximate the nominal FDR level of 5%, regardless of the dependency structure of the multiple tests (a tutorial review of the mass univariate analysis is provided by Groppe et al., 2011). This revealed ERP time points and their corresponding electrodes that were significantly different between the conditions.

Second, the ERP components that were identified to be sensitive to steering demands were submitted to permutation tests for each individual participant, in order to determine if these components were influenced by our difficulty manipulations for either disturbance bandwidth or control dynamics. A description of these single-subject permutation tests and their interpretation is provided by Maris and Oostenveld (2007). In brief, four key steps are performed for each participant: First, the selected electrode’s mean amplitude over the time-range of interest was computed for every trial. Second, these mean amplitudes were submitted to a one-tailed, paired-samples *t*-test to yield a test *t*-value. Third, a null-distribution of *t*-values was generated. All trials were pooled and randomly distributed (without replacement) to two subsets. A paired *t*-test was performed between these two sub-sets to generate a single *t*-value. This was repeated 10,000 times to generate a null distribution. Fourth, the test *t*-value was compared to this generated null-distribution to determine its *z*-value. An alpha-level of 0.05 was adopted to determine if the tested participant showed a significant difference for the difficulty manipulations. This procedure was repeated for each participant and each ERP component of interest.

## Results

### Steering Performance and Perceived Workload

Steering performance and the perceived workload were analyzed for our manipulations of steering demands. This was performed independently for our manipulations of disturbance bandwidth and control dynamics complexity with the use of a paired-samples *t*-test. This was to validate that our participants responded appropriately to our difficulty manipulations for “easy” and “hard”. An alpha-level of 0.05 was adopted for significance testing. The Cohen’s *d* is reported for the effect size. Overall, we found medium to large effects in our manipulations of difficulty for both performance and perceived workload.

Steering performance was evaluated based on the root-mean squared deviation of the rotating line from the reference line (i.e., RMSerror). The mean RMSerror was significantly higher in the “hard” than in the “easy” condition for manipulations of the disturbance bandwidth (*t*_(23)_ = −6.6, *p* < 0.001, *d* = −1.4) and control dynamics (*t*_(23)_ = −2.2, *p* = 0.04, *d* = −0.4).

**Figure 2:**
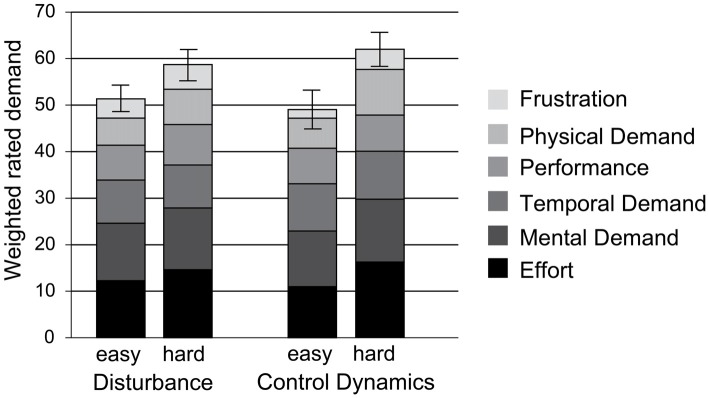
**Weighted sum of the six subscales of the NASA-TLX that were perceived by the participants as contributing to the overall workload.** The error bars represent the 95% confidence interval.

Perceived workload was based on the participants’ responses in the NASA-TLX questionnaire. The resulting workload score is the weighted sum of six subscales that were perceived by the participants as contributing to the overall workload in the following proportions: Effort: 24.5%, Mental Demand: 23.1%, Temporal Demand: 17.7%, Performance: 14.3%, Physical Demand: 13.4%, and Frustration: 7.0%. The “hard” condition was rated as being significantly more demanding than the “easy” condition for both manipulations (disturbance bandwidth: *t*_(23)_ = −3.4, *p* = 0.00, *d* = −0.7; control dynamics: *t*_(23)_ = −3.6, *p* < 0.001, *d* = −0.7). Figure 2 illustrates the distribution of the six subscales over the two manipulations and two levels of difficulty.

### ERP Results

This section is divided into three parts that describe the three analyzed aspects of the elicited ERP components. First, we present the comparison of the two distractor stimuli. Second, we present the results of the comparison between the viewing and steering trials. Third, we present the results of the comparison between the two applied manipulations of steering demands.

#### Comparison of the Two Distractor Stimuli

**Figure 3:**
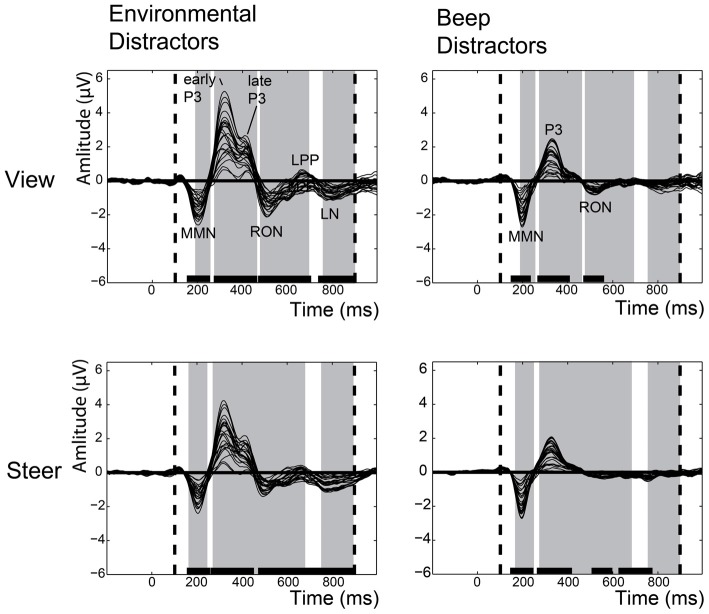
**Grand averaged waveform of the event-related potentials (ERPs) that were elicited by the environmental distractors (left column) and the beep distractors (right column), separately for the viewing (top row) and steering (bottom row).** The grand averaged waveform shows the difference wave between the ERPs elicited by the environmental/beep distractors and the standard beep-tones. Every line represents one electrode. The dashed vertical lines represent the time window of interest (100–900 ms). The black bars specify the time range when the ERP amplitudes were significantly different from the pre-stimulus time-interval. The gray areas highlight the time-periods where the ERPs of the beep and environmental distractor differed significantly from each other.

To begin, we separately identified ERP components that were elicited by the environmental and beep distractors. Therefore, we identified, with mass univariate analysis, the time-periods for which ERP amplitudes were significantly different from the pre-stimulus time interval. Figure 3 illustrates the grand averaged waveforms and indicates significant ERP components with black bars. The environmental sounds elicited, in the steering and the viewing condition, a MMN, an early and late P3, a RON, a late positive potential (LPP) and a late negativity (LN). The beep distractors elicited a MMN, a P3a that was not further discriminable for early and late P3 sub-components, a RON, and (only in the steering condition) a LN.

Subsequently, we contrasted the ERPs that were elicited by the environmental and beep distractors. This was performed separately for the steering and the viewing trials with the use of mass univariate analyses. Figure 3 highlights (in gray) the time-periods where the ERPs of the beep and environmental distractor differ significantly. This reveals that environmental distractors generate larger P3, RON, LPP and LN components than the beep distractors. The beep distractor generated an MMN that peaked earlier than the environmental distractor.

#### General Demands of the Steering Task

**Figure 4:**
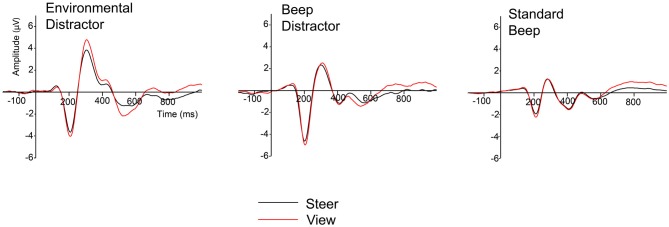
**Grand averaged waveforms after the stimulus presentation of the environmental distractors, beep distractors and standard beeps for the viewing (red) and steering (black) trials**.

Here, we determined the influence of steering demands on the elicited ERP components. In the grand averaged waveform (see Figure 4), the influence of the steering demands can be mainly observed in the ERPs that were elicited by the environmental distractor stimuli and to a lesser degree, in the beep distractors. As expected, for the ERPs that were elicited by the standard beeps the steering demands did not have a visible influence.

**Figure 5:**
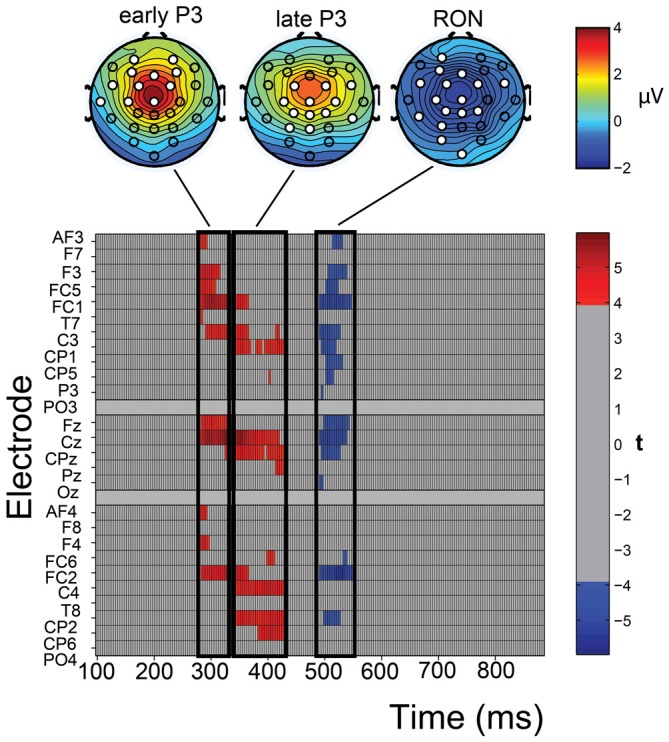
**The raster diagram (bottom) shows the comparison results of the environmental distractor ERPs across viewing and steering trials.** A mass univariate analysis analyzed every time point (256 Hz) between 100–900 ms for all 26 electrodes. Red/blue rectangles represent time points and electrodes where the difference between the ERPs in the viewing and steering trials was significantly positive/negative. Scalp topographies are provided (top) for the three significant time-intervals where significant differences were found. Scalp potential amplitudes are illustrated as heat maps and significant electrodes that differentiated between the viewing and steering conditions are marked white.

Using a mass univariate analysis, we determined the electrodes and time points for which ERPs were significantly decreased during the steering trials, relative to the viewing trials. This was performed separately for the ERPs that were elicited by the environmental distractors and those elicited by the beep distractors. The ERPs elicited by the beep distractors were not significantly influenced by steering demands for any electrode at any time point. In contrast, the ERPs elicited by the environmental distractors were selectively decreased by steering demands at specific time-points and electrodes. Figure 5 provides a raster diagram to indicate the time-points and electrodes where ERPs of the environmental distractors were sensitive to steering demands. The scalp topographies for significant ERP components are provided together with the significant electrodes, indicated as white filled circles. Altogether, we find that steering demands diminish an early and late sub-component of the novelty-P3, and the RON. These ERP components have a frontocentral distribution.

Steering demands significantly decrease the early P3 generated by the environmental distractor in the time window between 280–330 ms in the frontocentral electrodes (AF3, AF4, F3, F4, FC5, FC1, FC2, C3, T7, Fz, Cz). The late P3 was significantly decreased between 330–430 ms in the central electrodes (FC1, FC2, FC6, C3, C4, CP1, CP2, P3, CP6, Cz, CPz, Pz). Interestingly, steering demands influence late P3 amplitudes at electrodes that do not correspond with the frontal electrodes, which exhibit the largest late P3 amplitudes. The RON was significantly decreased in the time window of 500–550 ms over the left electrodes (AF3, F3, FC1, FC2, FC5, FC6, C3, CP1, CP2, CP5, P3, PO3, Fz, Cz, CPz, Oz).

**Figure 6:**
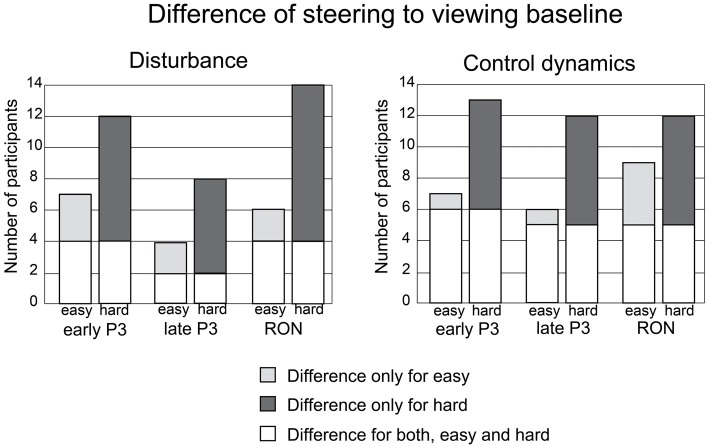
**Permutation tests were performed to evaluate steering manipulations of the disturbance (left) and control dynamics (right).** Bar plots indicate the number of participants that exhibit a significant difference in their early P3, late P3 and RON for the steering condition (“easy”, ”hard”) relative to the viewing baseline. White bars indicate participants who showed a reliable difference for both “easy” and “hard” conditions. Light/Dark gray bars indicate participants who showed a reliable difference for only the “easy”/“hard” condition.

Following this, we employed permutation tests to analyze the influence of steering demands on the early P3, late P3, and RON of individual participants, when elicited by environmental distractors. Single trials of the two steering conditions (“easy” and “hard”) were independently compared to the baseline viewing condition. For each participant, we submitted the recorded data from the electrodes and time points of the targeted ERP components to the permutation test. This was performed independently for the two different manipulations of steering difficulty, namely disturbance bandwidth and control dynamics complexity. Figure 6 plots the number of participants that produced significantly larger ERP amplitudes in the viewing compared to the “easy” or “hard” steering trials for the targeted ERP components.

The single-subject analysis produced results that were consistent across both manipulations (i.e., disturbance bandwidth and control dynamics complexity) and all three analyzed components (early P3, late P3 and RON). More participants showed a significant reduction in the three targeted ERP components for the “hard” condition than the “easy” condition, relative to the “viewing” baseline. Figure 6 also indicates differences across individuals, in terms of how they varied in response to the difficulty manipulations. White bars represent participants whose selected ERP components were diminished in both the “easy” and “hard” conditions. The dark gray bars represent participants whose ERP components were only diminished by the “hard” condition but not by the “easy” condition. The light gray bars represent participants whose ERP components were only diminished by the “easy” condition but not by the “hard” condition. Overall, the results are in line with our expectations. More participants whose ERPs were unaffected by the “easy” condition were, nonetheless, affected by the “hard” condition than *vice versa*.

#### Influence of the Steering Manipulations

**Figure 7:**
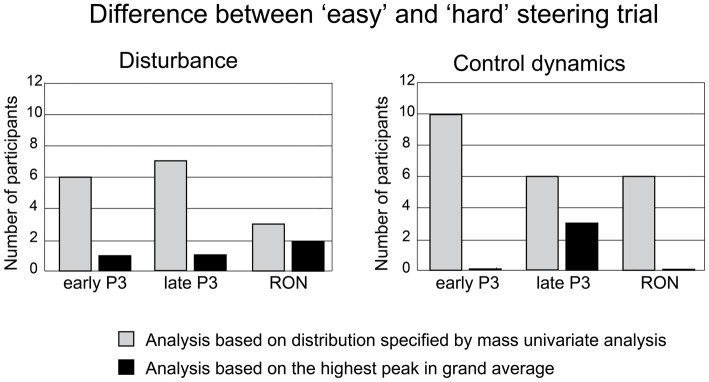
**Permutation tests were performed to evaluate steering manipulations of the disturbance (left) and control dynamics (right).** Bar plots indicate the number of participants that exhibit a significant difference in their early P3, late P3 and RON between the “easy” and “hard” conditions. Light gray bars indicate participants who showed a reliable difference between the “easy” and “hard” conditions when the analysis was based on the electrodes and time points indicated by mass univariate analysis. Black bars indicate participants who showed a reliable difference between the “easy” and “hard” conditions when the analysis was based on the peak in the grand averaged waveform.

Permutation tests were conducted to identify the number of participants who reliably exhibited lower amplitudes for the targeted ERP components (i.e., early P3, late P3, and RON) in the “hard” trials relative to the “easy” trials. Figure 7 represents these results as gray bars. The same analysis was performed based only on the peak-amplitude electrode and corresponding time-window (i.e., ±20 ms around the grand average peak). This is the approach that is employed by comparable research (cf., Miller et al., 2011; Dyke et al., 2015). Figure 7 represents these results as black bars. A comparison shows that a mass univariate analysis approach identified ERP components that were more sensitive to the current steering manipulations. Finally, more participants responded in the expected direction for the targeted ERP components when the complexity of the control dynamics was manipulated for difficulty than when the bandwidth of disturbance was manipulated.

## Discussion

The current study was designed to investigate if the demands of a steering task would attenuate the amplitudes of ERPs to task-irrelevant stimuli. It is in this regard that the current work sets itself apart from previous work that evaluated steering demands by measuring the ERPs to the task-relevant stimuli of a concurrent secondary task (e.g., Wickens et al., 1983, 1984; Sirevaag et al., 1989). The main findings of the current study are that steering demands can significantly reduce the amplitudes of three ERP components (i.e., early P3, late P3, and RON) of task-irrelevant auditory probes. However, this requires the probes to be complex environmental sounds and not simple beep-tones. Two aspects of the steering task (i.e., disturbance bandwidth and control dynamics complexity) were manipulated for steering demands and the found ERP components were significantly diminished in more participants during the difficult conditions relative to the easy conditions for both manipulations. The current results agree with a three-stage distraction model, whereby the ERP probes can be regarded as distractor stimuli that consume mental resources involuntarily (Schröger and Wolff, 1998; Escera and Corral, 2007; Wetzel and Schröger, 2014). Therefore, we will discuss our results within this simple framework. The discussion will be organized as follows. First, we shall discuss the differences between complex environmental sounds and simple beep tones in order to understand why the former elicit ERPs that are sensitive to steering demands while the latter do not. Second, we will discuss the implications of each ERP component that was found to respond to steering demands. Third, we will discuss the observed differences in the ERPs between manipulating either the disturbance bandwidth or the control dynamics complexity.

### Comparison of Complex Environmental Sounds and Beep-Tones Distractor Stimuli

Both types of task-irrelevant distractor sounds elicited a characteristic waveform that contained ERP components, which were significantly different from the baseline (see Figure 3). In temporal order, they are the MMN, the novelty-P3, and the RON. Respectively, they are claimed to represent the three subsequent stages of how users respond to distraction (Schröger and Wolff, 1998; Escera and Corral, 2007; Wetzel and Schröger, 2014): (1) detection of the unexpected stimulus; (2) orientation towards the stimulus; and (3) disengagement from the distractor to re-orient back to the steering task. In other words, infrequently presented sounds are preferentially processed by the brain in spite of being task-irrelevant, whether they are complex environmental sounds or beep-tones. Two other ERP components (i.e., LPP and LN) were also elicited, but were not sensitive to steering demands.

Environmental sounds elicited ERPs that differed from the beep tones in two ways. First of all, they elicited larger ERPs. Second, their ERPs contained components that were sensitive to steering demands. These two aspects are related. To begin, it can be argued that the larger novelty-P3 and RON amplitudes (see gray areas in Figure 3) indicate that environmental sounds recruit more corresponding mental resources than the beep sounds (Kok, 1990, 1997). This difference is apparent in the baseline viewing condition during which the participants’ mental resources were unoccupied and readily available. Involuntary resource recruitment is attenuated when participants are required to perform a steering task (i.e., in the steering trials), but only for the novelty-P3 and the RON of the environmental distractors (see Figure 5). This is because the steering task reduced the amount of available resources to a lower level than task-irrelevant environmental distractors would typically recruit. In view of this, we believe that our use of task-irrelevant environmental distractors is a more direct assessment of the resource demands of the steering task, when compared to dual-task paradigms that increase the resource demands of task-relevant stimuli that actively compete for resources with the steering task (Wickens et al., 1983, 1984; Sirevaag et al., 1989).

What are the properties of environmental sounds that allow them to recruit more mental resources and hence, generate larger ERPs even when they are task-irrelevant? Previous work suggests that distractor stimuli tend to recruit more resources if they are personally meaningful and/or exhibit high dissimilarity from their context. The personal meaning and dissimilarity from the context are respectively referred to as being stimuli specific and aspecific (Eimer et al., 1996; Hughes, 2014). Specific aspects are parameters that are inherent to the stimulus, which represent its meaning to the observer (Hughes, 2014). For example, one’s personal ringtone is more distracting, as reflected by larger elicited ERPs, than another person’s ringtone (Roye et al., 2007). In the current study, the environmental distractors represented familiar objects (e.g., dogs, cats, babies), which have more personal meaning than the beep-tone distractors. Thus, they can be expected to recruit more resources. Aspecific aspects of the eliciting stimulus recruit resources involuntarily due to its embedded presentation context. For example, a task-irrelevant female voice has been shown to be less distracting, as reflected by a decrease of performance in a visual recall task, when presented in a series of female voices than when presented in a series of male voices (Hughes et al., 2013). In the current experiment, we presented the environmental sounds as well as the beep sounds against a context of frequent beep tones. Arguably, environmental sounds that are a complex combination of multiple frequencies are more dissimilar to this context than their beep tone counterparts. This raised the likelihood that the environmental sounds would recruit more resources than their beep tone counterpart.

To sum up, task-irrelevant stimuli are more likely to be sensitive to task demands if they are personally meaningful and differ sufficiently from their embedded context. Some studies have been reported that have been successful in using task-irrelevant beep tones to evaluate task demands. However, these studies investigated complex tasks—that is, first person shooter (Allison and Polich, 2008) and racing games (Burns and Fairclough, 2015)—that, presumably, induced higher task engagement and varied in their resource demands at levels that beep tones were sensitive to. We expect the ERPs of task-irrelevant environmental sounds to be even more sensitive than beep tones to the resource demands of such complex tasks.

### Influence of Steering Demands on the Measured ERP Components

The current study is the first to employ task-irrelevant ERP probes in a task that allows for the systematic manipulation of different steering demands. Such task-irrelevant probes, in particular environmental sounds, continue to elicit ERPs with components that we have identified to be selectively diminished by steering demands: early P3, late P3 and RON (see Figures 3, 5). As noted before, these components correspond to the mid and late stages of a three-stage distraction model (Schröger and Wolff, 1998; Escera and Corral, 2007; Wetzel and Schröger, 2014). From the perspective of this model, steering demands did not inhibit our participants’ capacity for detecting unexpected occurrences. Instead, steering demands significantly diminished the extent to which available mental resources could be directed towards the processing of distractor stimuli. In turn, this hinders an efficient re-orientation away from the distractor stimuli. Altogether, these findings demonstrate that steering places demands on mental resources that would otherwise be directed towards an instinctive evaluation of unexpected events. These resources are based on attentional processes, but at a cognitive rather than a perceptual level. It is interesting to note that our participants were able to articulate this in that they rated the “hard” condition as being more demanding than the “easy” condition in terms of mental rather than physical effort (see Figure 2). This supports our research motivation in understanding the demands of a steering task beyond its perceptual and response requirements.

The ability to maintain an appropriate level for “distraction” is a fundamental capability of our attentional system and a critical aspect of effective vehicle handling. On the one hand, the capacity to be distracted by unexpected events is necessary when these events reflect potential dangers in the environment. For example, the phenomenon of “attentional tunneling” refers to scenarios when high-performance pilots miss unexpected hazards given their increased engagement with vehicle handling. Such undesirable instances have even been observed in novel cockpit environments that are designed to promote engagement with vehicle handling, for example when synthetic vision displays with intuitive flight guidance were employed for fixed-wing control (Wickens and Alexander, 2009). On the other hand, distraction presents a danger when it interrupts and prevents one to carry out a safety-critical task. In the United States, driver distraction raises the risk of a light-vehicle near-crash/crash to approximately three times of the baseline level (Klauer et al., 2006; Regan et al., 2011). Task-irrelevant or task-relevant probes can be judiciously employed in steering environments depending on whether the goal is to investigate either involuntary or voluntary distraction. A perspective that considers steering environments in terms of the driver’s engagement with the steering task and potential distractions (both voluntary and involuntary) is more likely to yield practical insights and operational recommendations than one that simply evaluates driving workload.

In this study, we show that both, early and late P3 components, were influenced by steering demands. These components are discriminable from each other in terms of their spatial and temporal characteristics. Functionally, the early P3 reflects a sensitivity towards violations of one’s model of the environment at a post-sensory stage (Ceponiene et al., 2004). The late P3 relates to the attending of the unexpected event itself, presumably for the purpose of updating one’s model of the environment when deemed necessary (Escera et al., 1998; Yago et al., 2003; SanMiguel et al., 2008). Earlier studies have provided mixed evidence on the relationship of workload and these components. Difficulty manipulations in a complex Tetris^®^ gaming environment have been found to only diminish early P3 amplitude (Dyke et al., 2015), while other studies, in particular those that target memory load, identified the late P3 as the only P3 sub-component that is influenced by workload (Escera et al., 1998; SanMiguel et al., 2008). Until the subtle interactions between workload and these P3 sub-components are better understood, we recommend employing approaches such as mass univariate analyses to determine the role of either sub-components in new task paradigms (e.g., steering), so as to reduce the risk of false positives.

Characterizing the relevant sub-components in terms of their spatial and temporal distributions provides an additional benefit. It allowed us to discriminate between manipulations of steering demands that would not be noticeable by only analyzing the peak, given inter- and intra-individual differences (cf., Munka and Berti, 2006; Miller et al., 2011; Dyke et al., 2015). In the current work, we show that more participants discriminated for the “easy” and “hard” steering trials compared to when the analysis was based on the highest peak in the grand average (see Figure 7). Mass univariate analysis also offers an additional benefit in that it more accurately defines the spatial location of the effect of interest. In the case of late P3, we find that the electrodes that are sensitive to steering demands have a more parietal distribution than the peak amplitude electrode. This agrees with the work of Yago et al. (2003) who also defined a discriminable parietal aspect of late P3 that is claimed to be involved with working memory updating and is believed to originate from the posterior and superior parietal lobes.

Besides early and late P3, we found that steering demands significantly decreased RON amplitude. RON is believed to reflect the re-orientation of attention from the distractor stimulus (Schröger and Wolff, 1998; Escera and Corral, 2007; Wetzel and Schröger, 2014). In this sense, it can be regarded as a disengagement of resources from processing distractor stimuli. Our results are comparable to those reported by Berti and Schröger (2003) who also found that increasing workload in the primary forced-choice task reduced RON amplitudes to a distracting task-irrelevant feature. In their experiment, participants were required to discriminate between sounds with “short” and “long” durations. Infrequent changes in the task-irrelevant pitch of the sounds produced RONs with an approximate latency of 500 ms. In their experiment, workload was manipulated either by allowing participants to respond immediately or by requiring them to respond upon the presentation of the next stimuli. The latter was considered to be more difficult as it involved a stimulus-response conflict. The amplitude of RON was found to be diminished in the difficult condition. Our current results indicate that a similar RON component can be diminished by increased task demands, even when the task is presented in a separate modality from the distractor. One reason for this could be that fewer resources were available to begin with, that could be effectively engaged by the distractor stimuli. Another reason could be that mental resources are more likely to be engaged with processing distractor stimuli for longer periods of time when sub-optimal levels of resources are allocated for their processing. In this case, the disengagement from the distractor stimuli could be expected to be less efficient. Whichever the reason, it is important to realize that RON reflects resource (*re*-)allocation processes at a post-sensory stage and that its amplitude does not simply decrease with increased workload. In fact, RON amplitudes have been found to be larger for the 1-back working memory task than its 0-back counterpart (SanMiguel et al., 2008). In this example, the 1-back task required participants to reference information of the primary task from recent history and larger RONs could have reflected a disengagement of resources from the distractor stimulus in addition to the re-allocation of resources to task-relevant information. We believe that our manipulation of steering demands resulted in decreased RON amplitudes because it only reflected the disengagement of resources from task-irrelevant distractor stimuli. If this is true, a dual-task paradigm that entails resource competition between a steering task and a task-relevant probe should result in larger RON amplitudes when steering demands are increased.

### The Steering Demands of Manipulating Disturbance and Control Dynamics

In the current study, we manipulated two aspects of steering that are known to influence steering demands—that is, disturbance bandwidth and control dynamics complexity. Both manipulations of steering difficulty had an influence on the identified ERP components in the expected direction (Figures 6, 7). Comparatively, this influence was evident in more participants when the complexity of control dynamics was manipulated. This result is in agreement with previous work that has shown a greater sensitivity of secondary task ERPs to the manipulation of control dynamics in the primary task (Isreal et al., 1980; Wickens et al., 1983, 1984; Sirevaag et al., 1989).

While encouraging, these results should be treated with caution. Our analyses reveal that our manipulations for steering demands do not influence the identified ERP components in all of our participants. In fact, some participants responded to steering demands only in the “easy” but not the “hard” condition, albeit to a lesser extent than *vice versa* (Figure 6). We believe that this reflects two aspects of inter-participant variance that are difficult to control for with the use of task-irrelevant ERP probes. First, the amount of resources that are involuntarily recruited for the processing of task-irrelevant probes. Second, steering competence and engagement with the steering task.

Participants can be expected to differ in terms of how meaningful they perceive different environmental sounds. Such differences could vary the extent to which these task-irrelevant distractors attract resources for their processing. If “insufficient” resources are recruited, changes in the level of available resources due to manipulations in steering demands can be expected to go undetected. To mediate this, future studies could consider employing environmental distractors that are not as easily recognizable. It has been shown that larger frontal and parietal novelty-P3s are elicited by environment sounds that are not as easily recognizable, compared to their more recognizable counterparts (Opitz et al., 1999). Moreover, it has been shown that the novelty-P3’s amplitude decreases with the repetition of familiar sounds but not unfamiliar sounds, presumably because participants are more effective in ignoring them (Cycowicz and Friedman, 1998, 2007).

Participants can be expected to vary in terms of steering proficiency. Therefore, some participants may only start to exhibit reduced levels of available resources under highly demanding scenarios. In fact, this is reflected in our results (see Figure 6). The current experiment employed fixed levels of steering difficulty. Subsequent studies could calibrate levels of steering difficulty for individual participants so that their performance discriminates sufficiently between “easy” and “hard” conditions. This would be similar to the use of adaptive methods in psychophysics to calibrate stimuli settings to individual differences in perception (Kingdom and Prins, 2010).

In spite of these limitations, our current findings are consistent with previous findings. The ERP components, which we have identified as being sensitive to steering demands, are more likely to differentiate for “easy” and “hard” conditions when disturbance bandwidth was manipulated than when control dynamics complexity was manipulated (cf., Isreal et al., 1980). This difference between the two manipulations is more prominent for early P3 and RON than it is for late P3. This suggests that increasing the complexity of the control dynamics limits how resources are directed towards and away from distractor stimuli.

### Conclusion and Outlook

To conclude, we have shown that the demands of a steering task influence how the brain responds to task-irrelevant stimuli. Specifically, steering demands diminish the amplitudes of the early P3, late P3, and RON that are elicited by task-irrelevant auditory distractors, which are personally meaningful and distinct from the background. A three-stage distraction model would suggest that steering demands decreases one’s sensitivity and likelihood to attend to unexpected events (early/late P3), as well as one’s capacity to re-orient back to the steering task at hand (RON). In particular, we found this to be true for steering manipulations that increased the complexity of control dynamics.

The three-stage model of distraction, and its associated ERP components, is a simplification. It assumes a serial chain of information processing of the distractor stimulus and is agnostic to how the stages could be selectively influenced by factors that do not pertain to the distractor stimulus itself. Thus, its explanatory power is limited. Our finding, that environment sound distractors are more “distracting” than deviant beep tones (and result in larger MMN, P3a, and RON), is in line with the predictions of the three-stage distraction model. However, the three-stage distraction model does not explain why steering demands selectively influence P3a and RON amplitudes but not MMN. In fact, there is accumulating evidence to suggest that dissociations exist between the three stages of distraction. Factors such as the predictability of the distractor, which is not dependent on the distractor *per se* but on the homogeneity of the sequence of stimuli that precedes it, can influence MMN and P3a but not RON (Horváth et al., 2008). Converse dissociations have been reported whereby increasing the predictability of an auditory distractor with a visual cue can decrease P3a and RON amplitudes but leave MMN intact (e.g., Sussman et al., 2003). Hence, more complex accounts have since been proposed that not only consider how distractor stimuli are processed but also how their processing might interact with the perceived regularity of the auditory scene (for example, see Bendixen, 2014). For now, it is sufficient to note that the demands of a steering task are reflected in how it modulates the distractibility of task-irrelevant environment sounds, as reflected in the early/late P3 and RON that they elicit. Besides electrophysiological responses, future experiments should be designed to investigate the behavioral consequences of distraction on steering performance (c.f., Parmentier, 2014). This could elucidate differences between distractor stimuli that passively reflect steering engagement and those that pose an involuntary conflict with the cognitive processes that underlie steering itself.

Task-irrelevant stimuli can be expected to be more easily integrated into real-world operations than the use of ERP probes that require an explicit response. In this regard, our current findings raise the opportunity of estimating steering demands across a wider range of scenarios than was previously considered to be practical. Furthermore, the use of task-irrelevant and task-relevant distractor stimuli can reveal complementary aspects of how mental resources are managed during steering. In this regard, they can be effectively employed to understand the demands of steering and users’ level of engagement with the steering task and their environment.

## Author Contributions

HHB and LLC: conception of the work; LLC and MS: design and interpretation of the data; LLC and MS: acquisition and analysis of the data; HHB, LLC and MS drafting and revision of the work; HHB, LLC and MS: final approval of the version to be published and agreement to be accountable for all aspects of the work.

## Funding

This research was supported by the German Research Foundation (DFG) within project C03 of SFB/Transregio 161 as well as by the Max Planck Society.

## Conflict of Interest Statement

The authors declare that the research was conducted in the absence of any commercial or financial relationships that could be construed as a potential conflict of interest.
